# Implementation of Visual Odometry on Jetson Nano

**DOI:** 10.3390/s25041025

**Published:** 2025-02-09

**Authors:** Jakub Krško, Dušan Nemec, Vojtech Šimák, Mário Michálik

**Affiliations:** Department of Control and Information Systems, FEIT, University of Zilina, 010 26 Zilina, Slovakia; dusan.nemec@uniza.sk (D.N.); vojtech.simak@uniza.sk (V.Š.); mario.michalik@feit.uniza.sk (M.M.)

**Keywords:** Jetson Nano, visual SLAM, visual odometry, ORB-SLAM3, single board computer

## Abstract

This paper presents the implementation of ORB-SLAM3 for visual odometry on a low-power ARM-based system, specifically the Jetson Nano, to track a robot’s movement using RGB-D cameras. Key challenges addressed include the selection of compatible software libraries, camera calibration, and system optimization. The ORB-SLAM3 algorithm was adapted for the ARM architecture and tested using both the EuRoC dataset and real-world scenarios involving a mobile robot. The testing demonstrated that ORB-SLAM3 provides accurate localization, with errors in path estimation ranging from 3 to 11 cm when using the EuRoC dataset. Real-world tests on a mobile robot revealed discrepancies primarily due to encoder drift and environmental factors such as lighting and texture. The paper discusses strategies for mitigating these errors, including enhanced calibration and the potential use of encoder data for tracking when camera performance falters. Future improvements focus on refining the calibration process, adding trajectory correction mechanisms, and integrating visual odometry data more effectively into broader systems.

## 1. Introduction

In recent years, significant advancements have been made in the development of visual SLAM (vSLAM), with a great deal of research focused on improving visual-inertial SLAM. These improvements address challenges such as motion blur, poor or repetitive textures, and occlusions. The most straightforward approach for integrating visual and inertial data is loosely coupled sensor fusion, where the IMU functions as an independent module within the vision-based system.

There are numerous algorithms available for visual odometry, each differing in terms of supported camera types, hardware requirements, precision, and compatibility with various hardware architectures (e.g., ×86, ARM). One of the initial steps in our implementation was selecting a suitable software library that met our specific requirements. Given that we were working with the ARM architecture, the chosen algorithm needed to be easily modifiable to accommodate the camera we had available.

After compiling the necessary libraries, the next objective was to develop a script to interface the algorithm with our selected camera. Additionally, several potential applications of visual odometry were explored, particularly in improving detection in challenging environments or mitigating issues such as motion blur and poor lighting conditions.

Our implementation was evaluated using the EuRoC dataset, which is freely available for download online. The algorithm’s performance was further validated by testing it on a robot equipped with a camera mounted on top. The robot’s wheel encoders provided ground truth data, which allowed us to compare this data with the output from ORB-SLAM3 and assess its accuracy in a real-world scenario [1].

## 2. Literature Review

A significant portion of our work was facilitated by the use of the ORB-SLAM3 library, which is capable of performing both visual and multi-map Simultaneous Localization and Mapping (SLAM) with monocular, stereo, or RGB-D cameras. One of the primary advantages of ORB-SLAM3 is its ability to integrate with an Inertial Measurement Unit (IMU) and support a multi-map system, which aids in spatial localization when the system loses track of its position and re-identifies keyframes from a previous map. This system is optimized for both indoor and outdoor environments and can operate in pure visual or visual-inertial modes. The algorithm utilizes Oriented FAST and Rotated BRIEF features, which give the system its name, ORB. Furthermore, ORB-SLAM3 supports loop closure, enabling the merging of maps when keyframes from different maps are spatially aligned, with one belonging to the active map. In this process, a welding window is constructed from keyframes, duplicate points are identified, and map graphs are merged [2].

Accurate vehicle localization is essential for mobile robots to achieve autonomous navigation, relying on various sensors and techniques like wheel odometry, INS, GPS, and visual odometry (VO). Each method has limitations: wheel odometry suffers from drift, INS accumulates errors, and GPS, though widely used, is ineffective indoors or in confined spaces and lacks the precision required for some applications. More advanced GPS systems like differential and real-time kinematic GPS can offer centimeter-level accuracy but are costly. Visual odometry techniques using camera images to estimate movement provide a promising approach to localization by extracting visual information from the environment [3].

In dense 3D SLAM, spatial mapping is achieved by fusing data from a moving sensor to construct surface representations, allowing for accurate, viewpoint-invariant localization and detailed scene interpretation. Most dense reconstruction approaches rely on RGB-D sensors, such as those used in the Kinect or Orbbec cameras. These systems achieve impressive results using Signed Distance Functions (SDF) for scene geometry representation and the Iterative Closest Point (ICP) algorithm for camera tracking [4].

Other SLAM solutions include SLAMCore, a commercial visual-inertial SLAM system that provides precise 3D positioning of vehicles without requiring expensive infrastructure. It also supports Real-Time Location Systems (RTLS) for enhanced positioning and correction, along with built-in object detection shared between vehicles to improve safety. However, a significant drawback of SLAMCore is its limited flexibility, as it cannot be easily modified beyond the initial configuration provided by the manufacturer. In contrast, open-source software offers greater adaptability, allowing for modifications tailored to specific use cases [5].

Another approach focuses on enhancing movement detection using lightweight techniques that can run on CPU-only devices, making it suitable for autonomous vehicles equipped with a single RGB-D camera. These systems generally fall into two categories: deep learning-based methods, which require a Graphics Processing Unit (GPU) for data processing, resulting in high accuracy but increased system cost due to the need for more computational power; and systems that run exclusively on a Central Processing Unit (CPU), which are more cost-effective but lack the accuracy required for reliable navigation in complex environments [6].

In recent years, advancements in LiDAR sensors have led to the development of V-LOAM (Visual-LiDAR Odometry and Mapping), based on the LOAM algorithm. This approach combines monocular feature tracking with IMU measurements to provide distance information for LiDAR scan matching. While the algorithm operates on a frame-by-frame basis and lacks global consistency, the authors propose a loosely coupled method that maintains a keyframe database for global pose graph optimization, thereby improving global consistency [7].

SLAM technology plays a fundamental role in robotic perception, enabling autonomous navigation and exploration of unknown environments. Typically, SLAM systems rely on sensors such as LiDAR, cameras, and IMUs to perceive their surroundings, constructing a consistent map of the environment and continuously updating the robot’s position within it. In this context, DIO-SLAM (Dynamic Instance Optical Flow SLAM) integrates instance segmentation with dense optical flow algorithms to distinguish between non-rigid, stationary, and moving rigid objects. When moving objects become stationary, their static features are retained for tracking, optimization, and map construction [8].

LOFF (LiDAR and Optical Flow Fusion Odometry) is predominantly used in unmanned vehicles. However, because optical flow estimates movement at the image scale rather than the real-world scale, it is typically used for target recognition and depth estimation rather than vehicle position estimation. LOFF integrates LiDAR SLAM with optical flow odometry to enhance the performance of traditional LiDAR SLAM, particularly in tunnel-like environments. This approach was tested on a quadrotor UAV equipped with a top-mounted LiDAR sensor, which provided 360-degree non-repetitive point cloud data for LiDAR SLAM. Although this technique is beneficial for aerial robots, it was not necessary in our case, as we employed a wheeled robot [9,10].

Similarly, in an article on measuring spatial pose using IMU and Apriltag technology, various methods for determining a robot’s pose relative to its workspace are discussed. While systems such as Vicon, Optitrack, and PTI Visualeyez provide accurate pose data, their high cost makes them unsuitable for educational or research purposes. Additionally, these systems rely on infrared (IR) light, which limits their use outdoors, as sunlight contains IR light that can interfere with sensor measurements. The paper emphasizes the advantages of fusing camera and IMU data, as visual odometry (VO) alone cannot provide real-time global pose information. Apriltags are used to obtain the relative pose of the camera to the tag, and an Extended Kalman Filter (EKF) is employed to reduce noise in the inertial data, thereby improving position accuracy [11].

By integrating deep learning techniques with visual odometry, features can be dynamically removed or altered to enhance the positioning accuracy and robustness of systems like ORB-SLAM3. A dynamic feature point detection and removal process was embedded in the tracking thread to eliminate the influence of dynamic feature points on camera pose estimation, significantly improving the accuracy and robustness of the SLAM system. Validation using TUM datasets demonstrated that the improved algorithm resulted in substantial gains in localization accuracy and detection robustness [12].

The task of matching visual data, which includes local estimates of orientation and path overlaps, with a hand-drawn map is more complex than simple time warping. Visual odometry captures the local path structure but accumulates global errors, making direct global matching between visual and map-based orientations ineffective. Instead, the approach involves matching video frames with map points based on local orientation differences, specifically focusing on rotations around the vertical axis. The matching process is formalized through a function that maps discretized map points to video frames, with criteria ensuring smoothness, minimal local orientation differences, and proximity-based frame matching.

A graphical model is used to encode these constraints, with edges representing monotonicity, orientation consistency, and proximity in three edge types. To solve this, a loopy belief propagation method is employed to maximize the match probability across these constraints. Given the loops in the model, a dynamic programming approach cannot be used, so an asynchronous message-passing scheme is applied to improve convergence. This method produces more accurate matches compared to dynamic programming, as shown by the comparison of matches, particularly in areas where the path bends [13].

Ultimately, we chose to use ORB-SLAM3 due to its simplicity and lower resource requirements, as it can function with only a camera and does not necessarily require an IMU. This version, compared to its predecessors, introduces support for multi-map atlases, allowing the creation of new maps when tracking is lost and merging these maps when similar points are detected. One modification we made involved compiling the source code on an ARM platform and developing custom software to interact with the camera.

## 3. Hardware Architecture and Implementation

The initial steps for implementing the ORB-SLAM3 algorithm involved compiling the essential libraries required for its operation, including OpenCV [14], Pangolin [15], Eigen [16], and DBoW2 [17]. OpenCV, an open-source computer vision library, processes images captured by the camera. Eigen simplifies matrix and vector operations by providing methods in header files, while DBoW2 is used to index and convert images into a hierarchical representation, facilitating the approximate search for similar image patterns.

The system was evaluated using the EuRoC dataset, which consists of 11 sequences that form a map and are available in various configurations such as monocular, stereo, and stereo-inertial. The stereo-inertial configuration has been shown to be three to four times more accurate than algorithms like Kirmera or other visual odometry methods. The EuRoC dataset provides ground truth for each sequence relative to the IMU body reference frame, with visual algorithms reporting trajectories centered on the left camera.

An alternative dataset for evaluating accuracy is the TUM-VI dataset, which was recorded using two fish-eye cameras and an inertial sensor. In contrast, the EuRoC dataset employs pinhole cameras along with an inertial sensor. However, the TUM-VI dataset offers ground truth data only in the room where all sequences begin and end, meaning that the error is measured by the drift at the sequence’s conclusion [2].

Hardware requirements for the algorithm depend significantly on the desired precision, as specified in its configuration file. Key parameters influencing this precision include the camera’s frames per second (FPS), the number of features extracted from each image, and the number of levels in the scale pyramid. Another critical factor affecting overall performance is the hardware used. During development, the algorithm was tested on a computer with an Intel Core i7 processor. However, for our implementation, we used a Jetson Nano, which offers significantly lower performance than the aforementioned CPU. Despite the reduced processing power, sacrificing some performance for lower energy consumption can be advantageous, especially when the algorithm is deployed on a battery-powered robot with limited energy capacity.

When using low-cost or low-power hardware, careful selection of software libraries is crucial, as some libraries may be poorly optimized, leading to higher hardware utilization and inferior results. In our testing, for example, OpenCV struggled to achieve more than 2 FPS, while the Orbbec SDK managed 30 FPS without issues, simultaneously processing image data with ORB-SLAM3 in a separate thread [1].

### 3.1. Implementation

During the implementation phase, several challenges arose, particularly with the Jetson Nano, which struggled to compile certain libraries, operating near its performance limits. To establish a connection with the camera, we developed a custom C++17 script that integrated the compiled libraries with the Orbbec SDK v1.9.3, allowing the camera images to be processed and fed into the visual odometry algorithm. Initially, we considered using the OpenCV v4.8.0 library for this task, but it had a hard-coded timeout for camera reads, which resulted in stuttering images, rendering it unsuitable for the selected algorithm. Additionally, calibrating the camera was essential to minimize image distortion.

The Jetson Nano was selected primarily due to its small form factor, which facilitates integration into robotic systems, and its lower energy consumption compared to traditional computers, allowing it to function efficiently on battery power. However, proper power supply is critical to avoid potential crashes or SD card corruption, which can disrupt the entire process. Another important consideration was the ARM architecture, which offers better energy efficiency than x86-based systems. Despite these advantages, the Jetson Nano’s limited computing power led to difficulties during the compilation of larger libraries such as Pangolin and OpenCV, which occasionally caused the process to crash and required restarting. This issue further highlighted the need for a stable power supply to avoid system instability.

The created script consists of the initialization of the file for writing the acquired keypoints, algorithm objects for ORB-SLAM3 and objects for the Orbbec Astra Pro camera using OrbbecSDK. The script later needed to be optimized in such a way that there would be as little delay between capturing the frames and processing them as possible. This involved making the loops running inside the threads perform only the necessary tasks, and ensuring that the remaining tasks should be completed before they are started via mutex init, or checking if the camera is present.After locking the mutex and capturing color and depth frames, it was needed to convert them into a CV2 matrix and then set corresponding frags to indicate to the main thread that the variable for this image contains a new frame. When both these operations are finished, mutex is unlocked, and the main thread can process new images. The main thread waits until flags for both color and depth image are set and then locks the mutex for reading its content, and after processing both images and generating new position based on the releases, mutex and both flags are reset. This repeats until the command for shut down is received, which in this case is just a simple CTRL + C key press.

For the camera, we chose the Orbbec Astra Pro, an RGB-D camera that captures both color (RGB) and depth data. This dual capability simplifies distance estimation and obstacle detection, making it easier to measure the robot’s movement between keyframes. Additionally, the depth data enabled ORB-SLAM3 to generate a real-time 3D map of the environment, enhancing the system’s functionality [1].

The source code for the visual odometry algorithm is available on GitHub under the GPLv3 license, allowing free use for non-commercial purposes. The repository includes source files for external dependencies such as DBoW2, Sophus, and g2o, along with their respective makefiles to simplify the compilation process. It also contains a comprehensive ORB vocabulary with over a million descriptors for converting image features into a set of visual words. Furthermore, the repository provides example code for various camera types and evaluation scripts for testing the algorithm’s accuracy with datasets recommended in the README file [18].

### 3.2. Camera Interface

The initial version of our script utilized the OpenCV library for image acquisition in conjunction with the visual odometry (VO) algorithm, all within a single thread. However, this setup proved inefficient due to the bottleneck created by the image processing on the OpenCV side. To address this, we split the tasks into two separate threads, which initially improved performance. Unfortunately, after some time, the camera feed began to lag. We discovered that the root cause was a hard-coded timeout in OpenCV’s camera read method, which was slowing down the image acquisition process. To resolve this, one option would have been to modify the OpenCV library and adjust the timeout dynamically based on the system’s needs, but we opted against this approach. Instead, we transitioned to the OrbbecSDK, which is specifically designed for the Orbbec camera and avoids using the Linux V4L2 driver for camera access.

The OrbbecSDK employs a different method for camera access, simplifying integration with multi-threaded applications by handling image acquisition internally. The developer simply needs to capture the image from the provided pipeline and convert it into the required format. In our case, it was still necessary to use the OpenCV library, as ORB-SLAM3 requires the input image in matrix form, and it cannot process the raw image format provided by the SDK.

To ensure thread-safe operations and prevent data corruption, we implemented a mutex to manage access between the threads. The mutex locks the image data while it is being read and processed by the image acquisition thread, preventing the VO algorithm from accessing incomplete or corrupted images. Once the image thread finishes processing and capturing the data from the camera, the mutex is unlocked, allowing the VO algorithm to proceed with its computations [19].

### 3.3. OpenCV vs. OrbbecSDK

The choice of libraries largely depends on the camera type being used, as certain cameras may not function optimally with generic drivers and require specialized libraries to access their advanced features. This is why we opted to use the OrbbecSDK instead of the OpenCV library. For instance, the sample code for monocular depth cameras included with the ORB-SLAM3 algorithm uses an Intel RealSense camera, where the manufacturer’s SDK is employed for similar reasons. A significant advantage of the Intel library is that it automatically handles the conversion of images into OpenCV format, eliminating the need for manual intervention.

When comparing these libraries in terms of ease of use, OpenCV stands out as the more user-friendly option due to its extensive reference documentation, which provides detailed guidance on methods and functionalities. In contrast, OrbbecSDK requires a deeper understanding of the source files, as its documentation is less comprehensive. However, the sample files provided in the OrbbecSDK repository offer a valuable starting point, enabling users to adapt them to their specific needs despite the lack of extensive documentation.

### 3.4. Camera Calibration

There are several widely used methods for calibrating camera sensors, with the AprilTag and chessboard patterns being among the most common. The chessboard pattern is more widely adopted due to its simplicity, as it only requires a camera for calibration. In contrast, the AprilTag pattern requires the integration of Inertial Measurement Unit (IMU) sensors because the relative motion between individual tags is recorded and utilized to calibrate the IMU.

IMUs, while capable of providing real-time spatial information in six degrees of freedom (6 DOF), are prone to accumulated errors over time. AprilTags, however, offer higher localization accuracy by allowing the precise calculation of the 3D position relative to the camera. The drawback of AprilTags lies in their slow update rate, which hampers their real-time performance. The most effective approach to achieving precise positioning with a higher refresh rate involves using AprilTags for IMU data correction to reduce accumulated drift and improve overall accuracy [11].

**Figure 1:**
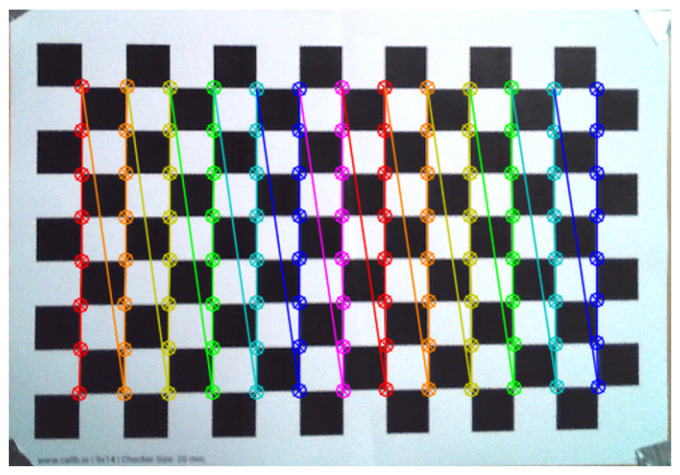
Chessboard pattern with highlighted corners [1].

In Figure 1, the detected corners of the chessboard pattern are highlighted, and their positions were used to compute the image distortion matrix, which was subsequently utilized in the ORB-SLAM3 configuration file. The values obtained through this process are unique to each camera, as sensor imperfections result in slight variations even among cameras of the same model. Consequently, the computed camera matrix can only be applied to the specific calibrated camera due to variations in lens distortion between different units of the same model.

The derived camera matrix is structured as follows:

(1) $$C_{m}=\left[\begin{array}{ccc} f_{x} & 0 & c_{x}\\ 0 & f_{y} & c_{y}\\ 0 & 0 & 1 \end{array}\right]\to \left[\begin{array}{ccc} 514.71 & 0 & 313.16\\ 0 & 514.73 & 236.05\\ 0 & 0 & 1 \end{array}\right]$$

The values in the matrix (Equation (1)) were obtained by detecting the corners of the chessboard pattern using OpenCV’s *cv.findChessboardCorners* function. The corners were then visualized using *cv.drawChessboardCorners*. Calibration images were converted to grayscale, and the detected corners’ coordinates were fed into the *cv.calibrateCamera* function, which computed the distortion coefficients and returned the camera matrix shown above. The calibration process was performed using a Python 3.9 script that captured around 30 images of the chessboard pattern from various angles and positions within the camera’s field of view. The resulting camera matrix contained the focal lengths ($f_{x},f_{y}$) and optical centers ($c_{x},c_{y}$) [1].

## 4. Experiment Results and Evaluation

This type of visual odometry algorithm cannot be effectively tested in a static environment, as it requires dynamic movement to generate meaningful data for processing. The algorithm relies on capturing sequential images from the camera as it moves through space, allowing it to track changes in the environment and estimate the camera’s motion. For this reason, testing must be conducted by either mounting the camera on a mobile platform, such as a robot, or manually moving the camera through the environment.

Static testing would not provide the necessary variation in perspective or changes in the visual scene that the algorithm needs to compute motion, detect keyframes, or build a map. By moving the camera, the algorithm can track features, estimate depth (in the case of RGB-D or stereo cameras), and generate the visual odometry necessary for accurate localization and mapping.

In dynamic tests, especially when mounted on a robot, the algorithm can be evaluated under realistic conditions that involve motion blur, lighting changes, and various types of environments (e.g., indoor vs. outdoor). Additionally, moving the camera allows the system to detect and correct errors in its localization through techniques such as loop closure, where it revisits previously seen areas. Therefore, dynamic testing is essential to properly evaluate the algorithm’s performance and accuracy.

### 4.1. Verification with Dataset

After successfully compiling the source files for the ORB-SLAM3 algorithm, we verified its functionality by running a test script that used the EuRoC dataset, which consists of images captured by a drone, to generate a map. The output of this test consisted of two files containing key points corresponding to the generated maps from the dataset. The repository also included a script designed to compare these generated key points with the ground truth data, allowing us to identify any discrepancies.

The resulting precision from the dataset testing showed a difference in path estimation ranging from 3 to 11 cm compared to the ground truth. This level of discrepancy is understandable, as the dataset was created using a well-calibrated camera under ideal conditions. These results suggest that the ORB-SLAM3 algorithm performs effectively when provided with accurate, high-quality input data.

Following the successful dataset testing, we proceeded with real-life testing by mounting the camera on a robot to assess the algorithm’s performance in more practical, less controlled conditions. While the authors of the algorithm did not specify expected accuracy values, deviations of up to 11 cm from the provided ground truth are generally considered negligible, especially given the complexity and variability of real-world environments. This suggests that the algorithm remains reliable for practical applications, despite minor inaccuracies. Additionally, compared to our own testing, they also used an IMU unit to capture movement data, which could help with mitigating discrepancies in case a sequence of images from the camera had some flaw in it (rapid movement resulting in blurry image, less ideal lighting conditions, etc.).

**Figure 2:**
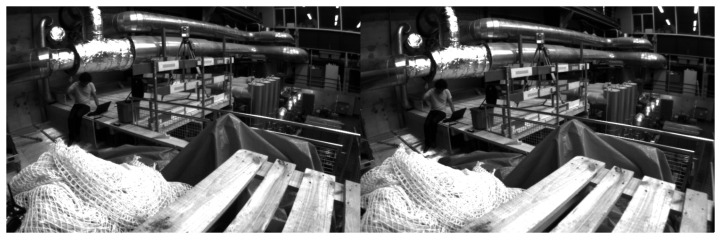
EuRoC dataset sample [20].

Each sequence in the dataset comprises of two grayscale images (Figure 2), with each image corresponding to one lens of the stereo camera utilized for data capture. The output from this test resulted in files that contain the coordinates of the trajectory points recorded during the mapping process.

**Figure 3:**
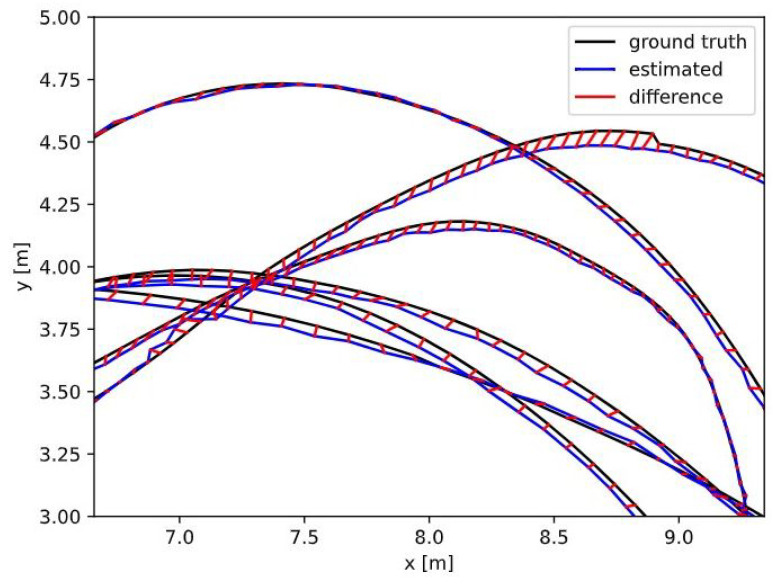
Detail of difference between estimated and ground truth trajectories [1].

After obtaining files with key points after running the algorithm with the mentioned dataset, the author provided a script which we could use to verify if the resulting data are correlating with the ground truth which was part of the EuRoC dataset. The output from this step was a graph that showed the estimated, ground truth and difference between these two trajectories (Figure 3). The mean difference between the trajectories was 3 cm, which is highly accurate since the dataset was created in ideal conditions [20].

### 4.2. Robot Testing

**Figure 4:**
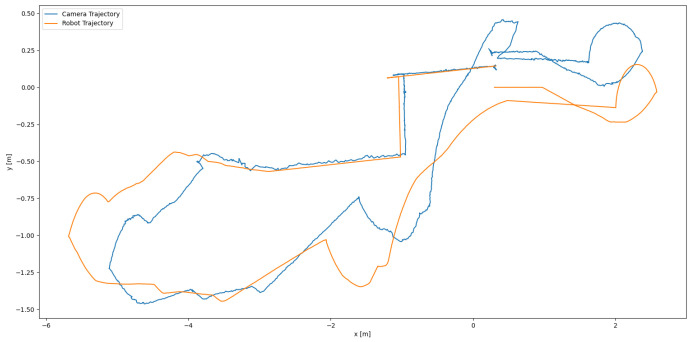
Comparison between robot and camera trajectories [1].

A significant advantage of using a robot to test the accuracy of the algorithm was the ability to compare the odometric data from the robot with the data generated by ORB-SLAM3. This comparison revealed noticeable differences, primarily due to encoder drift in the robot’s wheels. The measured trajectories are depicted in Figure 4, where the starting point is located at the closest proximity between the two trajectories. Generally, visual odometry is considered more accurate; however, this does not hold true under certain conditions, such as improper camera calibration, poor lighting, or uniform surroundings where distinguishing between individual features becomes challenging (e.g., plain walls, repeating patterns).

The deviations observed in the robot’s trajectory were caused by wheel slip, which depends on the surface type and the tires used. For this experiment, classical rubber tires were chosen, as they performed well both outdoors and indoors. Although omni-directional (Mecanum) tires were available, they exhibited poor traction on the tiled floor during the tests, causing tire slip and distorting results as the data from robot’s odometry featured discrepancies and the distance travelled by the robot did not match the distance travelled in the real world.

Before evaluating the accuracy of the algorithm, it was essential to offset the robot’s position. The robot’s position was recorded in relation to its centre, while the camera was mounted at the front. Consequently, the values would not align correctly without applying an offset.

The calculations for the new coordinates are as follows:

(2) $$x_{new}=x+d\cdot \cos\left(yaw\cdot \frac{\pi }{180}\right)\;\left[m\right]$$

(3) $$y_{new}=y+d\cdot \cos\left(yaw\cdot \frac{\pi }{180}\right)\;\left[m\right]$$

In these equations, *d* represents the distance from the robot’s centre to the camera (0.3 m), while *yaw* is the rotation angle at a given point, converted to radians. The variables *x* and *y* denote the current coordinates being adjusted.

The output file from the algorithm contains keyframes in a space-separated format, including the Linux timestamp, 3D position data (x, y, z coordinates), and 3D orientation represented as quaternions. To process this data, a Python script was created to parse each line and extract 2D position information, as the z-dimension was not utilized in this scenario. The pandas library in Python proved to be an excellent choice for managing larger data files.

After parsing the data, one of the trajectories needed to be offset so that both started from the same location, facilitating a more straightforward comparison. The achieved accuracy of the algorithm in this instance was approximately 88 cm. This discrepancy could be attributed to various factors, such as poor lighting conditions or camera calibration issues. Notably, when the robot was stationary, it exhibited slight oscillation in place, with a deviation of only 2 cm, which is minimal compared to the overall accuracy of the entire traveled trajectory.

**Figure 5:**
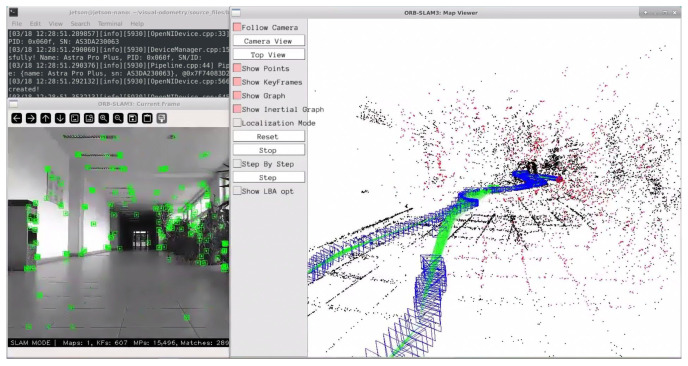
ORB-SLAM3 GUI [1].

The chosen algorithm also includes a graphical user interface (GUI) that provides a live view of the created map (Figure 5) alongside the current frame from the camera, with highlighted detected keypoints. On the right-hand side of the Figure 5 is a representation of created map. Blue rectangles show the positions of camera as the map was created, green points visualize trajectory of the robot and black points are detected features of environment (obstacles, walls, etc.). Obstacles are this accurate thanks to the depth sensing of the used camera, where it projects array of points into space and then measures how much this pattern is warped and by using this information it can visualize nearby objects. The number of keypoints can be defined in the configuration file, which is passed as an argument when starting the algorithm, along with the path to the vocabulary (VOC) dictionary that contains the descriptors. Optional arguments include the name of the output file and additional configuration flags, which vary depending on whether the original source files were used or if a customized version was created to meet specific needs.

**Figure 6:**
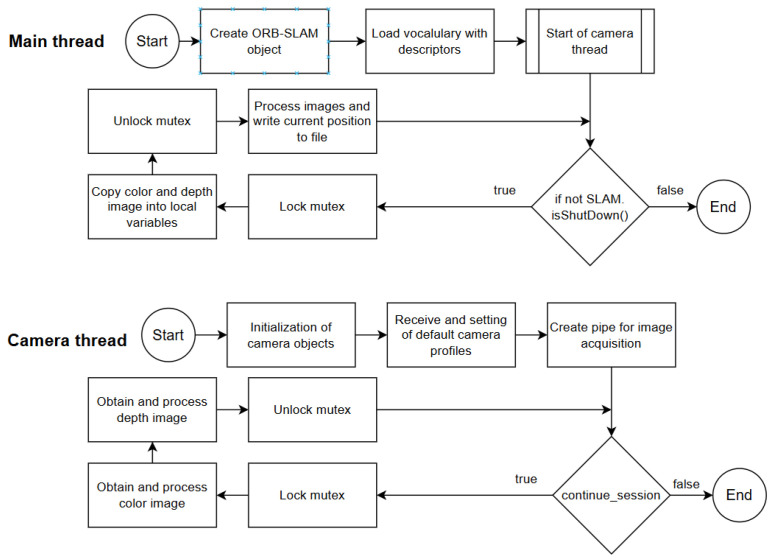
State-chart for implemented algorithm [1].

In Figure 6, the overall system diagram is visualized. It describes what happens after the created script is executed. Firstly, necessary objects for ORB-SLAM are created, then the camera is initialized and its capture thread is started. Exchange of images between threads is performed with the help of the mutex variable to prevent the overwrite of the variable from another thread while the current thread is currently using it. Without mutex usage, if both threads had access to the variable at the same, this could lead to memory corruption and possibly a runtime crash. When loops in both threads are active, at the start of each iteration, it checks if the given condition (*continue_session, SLAM.isShutDown*) is true, and if it is not then the loop is terminated and the program exits.

## 5. Discussion

In comparison to other approaches utilizing stereo cameras and IMU sensors for visual odometry, our methodology stands out due to its relative simplicity. We only needed to calibrate the camera, which is generally easier than calibrating IMU sensors. The majority of the camera calibration process can be accomplished in software using captured images. Conversely, calibrating an IMU involves determining various offsets while the unit is stationary and under different orientations, including upside-down positioning.

Our implementation has produced usable results for a small-scale use case, but there remains ample room for improvement. One area for enhancement is fine-tuning the camera calibration constants to ensure greater accuracy in depth perception. Additionally, we encountered challenges with repeating textures, which can lead to algorithm crashes when it processes NaN (Not a Number) data. To address this, we could incorporate motor step values from encoders mounted on the robot’s wheels. These values could serve as a backup when camera tracking fails, temporarily replacing camera data until tracking is restored. While the encoder data may be less precise—due to issues like wheel slip resulting in miscounted movements—it can still provide valuable backup under specific conditions.

Table 1 describes differences between testing with the provided dataset and by using data from the camera while creating a map in real-time. ATE stands for absolute trajectory error and describes simple differences between the estimated trajectory and ground truth after it has been aligned so that it has minimal error. A bigger value in robot testing is caused by wheel drift over time. RPE stands for reward pose error, which is the difference between the discounted predicted reward at the current state and the discounted predicted reward plus the actual reward at the next state. This value was present in small deviations from the path when the robot was changing its direction of travel, and rotation was differently processed by the algorithm compared to encoders on wheels. OEE stands for orientation estimation error, which refers to the inaccuracies in estimating the orientation of the robot or sensor within its environment. Accurate orientation estimation is crucial for effective mapping and localization because it affects how spatial relationships between the robot and its surroundings are perceived and utilized. When these parameters are compared against algorithms, we can clearly see that DynaSLAM is more accurate based on some parameters and in others it is less accurate.

Furthermore, the final detection accuracy could be enhanced by adding an option to manually offset the current position in cases of incorrect detection, or when keypoint tracking is lost due to monotonous environments. One potential solution for this problem is to implement automatic trajectory correction based on the last received keypoint locations or by reverting to the last known good values from the algorithm.

Additionally, developing a more robust script for converting raw data from the algorithm into trajectories would facilitate smoother integration into other software for further analysis or processing. This could improve overall usability and make the results more accessible for various applications.

### 5.1. Improving Accuracy

There are several strategies aimed at enhancing the accuracy of visual odometry (VO) on low-power hardware platforms.

Deep learning methodologies have demonstrated significant potential in improving VO accuracy while adhering to the constraints of Size, Weight, Area, and Power (SWAP). By utilizing model compression techniques and hardware acceleration, these approaches offer scalability and consistency across a range of aerial robots [22]. Furthermore, the integration of binary feature descriptors with Locality Sensitive Hashing facilitates efficient nearest-neighbor searches for real-time 6D pose recovery. This approach is particularly advantageous for resource-constrained devices such as mobile phones [23].

Several techniques address specific challenges associated with VO. For instance, an algorithm designed to directly utilize edge pixels has proven effective in handling low-texture environments, which are typically challenging for many state-of-the-art VO methods [24]. Additionally, the use of Vector Symbolic Architecture (VSA) as an abstraction layer has been explored to create VO algorithms optimized for neuromorphic hardware, achieving competitive performance on 2D VO tasks while maintaining low power consumption [25].

In summary, improving VO accuracy on low-power platforms involves the integration of multiple strategies. These include sensor fusion [22,26], optimization for specific hardware architectures such as GPUs [26], and addressing environmental challenges like motion blur and low-light conditions. The fusion of inertial measurements with vision systems [22], as well as the use of omni-directional cameras [26], has also shown considerable promise in enhancing VO accuracy without significantly increasing computational demands.

Stereo cameras are frequently employed in VO applications due to their ability to provide depth information, which enhances algorithmic accuracy. Gaoussou and Dewei (2018) propose a stereo camera-based approach that combines both feature-based and feature-less techniques for accurate 3D environment reconstruction [27]. Such stereo setups offer advantages for VO algorithms by capturing depth cues. Additionally, Vargas and Kurka (2015) describe the use of stereoscopic images for real-time VO processing in outdoor robotic navigation, further highlighting the benefits of stereo systems [28].

Nevertheless, monocular cameras remain widely adopted in VO systems due to their simplicity and lower hardware requirements. Scannapieco et al. (2019) discuss the implementation of monocular VO on small aerial platforms, acknowledging the limitations posed by environmental conditions such as varying illumination, which can reduce the effectiveness of monocular systems [29]. To mitigate these challenges, the integration of radar odometry with monocular VO is suggested to improve performance in sub-optimal lighting conditions.

The selection of a camera system is closely tied to the specific VO algorithm and available processing capabilities. For example, Ansorregi et al. (2021) compare ORB-SLAM with deep learning-based approaches like DF-VO in monocular VO systems, finding both effective in outdoor environments but constrained in indoor settings [30]. Nitin et al. (2023) further evaluate various VO methods using both monocular and stereo camera configurations, concluding that DF-VO performs optimally for short-range tasks with a monocular setup, while DROID-SLAM, when paired with stereo cameras, proves more effective for long-term localization tasks [31].

### 5.2. Thread Analysis

Jetson Nano is single board computer which has a four-core ARM processor and 4 GB of available RAM along with 128 CUDA cores in its built-in GPU. This makes it sufficient for most of the tasks, but for some tasks it could be restricting in the terms of computational power. In our use case it struggled a bit while compiling necessary libraries, but that was expected. In addition, it sometimes had problems when processing images from camera as its internal USB controller can be easily overloaded, which causes longer access times to connected devices. This was because this type of development board has no built-in Wi-Fi module, so we needed to add one to its USB port. Overloading of the USB controller could be caused by the fact that while processing data from the camera, Jetson needed to send this data to the computer running NoMachine software 8.9.3 for visualization purposes during the program runtime (Figure 5).

## 6. Conclusions

The primary objective of this paper was to outline the process of implementing visual odometry on the Jetson Nano without relying on Docker or ROS platforms as foundational elements at the operating system level. This endeavor proved to be relatively straightforward; however, we encountered some challenges related to supporting libraries and the hardware limitations of the chosen single-board computer.

To address the issue with supporting libraries, we replaced one with another that provided improved performance for the algorithm. Nevertheless, there are still opportunities for enhancement, such as refining the camera calibration to achieve better accuracy or utilizing an IMU or motor encoders to compensate for movement through monotonous environments. In such scenarios, the camera may fail to detect movement due to insufficient texture variation, which can lead to crashes in the algorithm.

Regarding the choice of algorithm for this task, we opted for a suitable vSLAM library that was easy to set up and relatively simple to integrate into a custom script, allowing compatibility with various cameras beyond those used in the library’s original testing. A critical consideration in this approach was the need to separate image acquisition and the algorithm into multiple threads to prevent them from blocking each other during execution.

The outcome of this research was a tailored implementation of the ORB-SLAM3 algorithm on the Jetson Nano, accompanied by a customized script that utilized a different camera driver than the original, specifically for the Orbbec Astra Pro camera. To ensure the camera operated correctly, we calibrated it using a chessboard pattern to mitigate lens distortion and achieve more precise tracking results and depth measurements of the surrounding environment. This calibration process was vital for enhancing the overall performance and reliability of the visual odometry system.

## Figures and Tables

**Table 1:** Error values of chosen algorithm.

Dataset	ATE [m]	RPE [m]	OEE [%]
EuRoC	0.03	0.01	2.5
Robot testing	0.11	0.05	4.5
DynaSLAM [21]	0.015	0.11	2.5

## Data Availability

No new data were created or analyzed in this study. Data are contained within the article.

## Abbreviations

The following abbreviations are used in this manuscript:

IMU	Inertial Measurement Unit
INS	Inertial Navigation System
LiDAR	Light Detection and Ranging
RTLS	Real-Time Location System
SDK	Software Development Kit
VIO	Visual-Inertial Odometry
